# Feasibility of an accelerated 2D-multi-contrast knee MRI protocol using deep-learning image reconstruction: a prospective intraindividual comparison with a standard MRI protocol

**DOI:** 10.1007/s00330-022-08753-z

**Published:** 2022-04-07

**Authors:** Judith Herrmann, Gabriel Keller, Sebastian Gassenmaier, Dominik Nickel, Gregor Koerzdoerfer, Mahmoud Mostapha, Haidara Almansour, Saif Afat, Ahmed E. Othman

**Affiliations:** 1 Department of Diagnostic and Interventional Radiology, Eberhard Karls University Tuebingen, Hoppe-Seyler-Strasse 3, 72076 Tuebingen, Germany; 2 MR Applications Predevelopment, Siemens Healthcare GmbH, Allee am Roethelheimpark 2, 91052 Erlangen, Germany; 3 Digital Technology & Innovation, Siemens Medical Solutions USA, Inc., Princeton, NJ USA; 4 Department of Neuroradiology, Langenbeckstraße 1, 55101 Mainz, Germany

**Keywords:** Magnetic resonance imaging, Deep-learning reconstruction, Image processing, Diagnostic imaging, Knee

## Abstract

**Objectives:**

The aim of this study was to evaluate the image quality and diagnostic performance of a deep-learning (DL)–accelerated two–dimensional (2D) turbo spin echo (TSE) MRI of the knee at 1.5 and 3 T in clinical routine in comparison to standard MRI.

**Material and methods:**

Sixty participants, who underwent knee MRI at 1.5 and 3 T between October/2020 and March/2021 with a protocol using standard 2D–TSE (TSE_S_) and DL–accelerated 2D–TSE sequences (TSE_DL_), were enrolled in this prospective institutional review board–approved study. Three radiologists assessed the sequences regarding structural abnormalities and evaluated the images concerning overall image quality, artifacts, noise, sharpness, subjective signal-to-noise ratio, and diagnostic confidence using a Likert scale (1–5, 5 = best).

**Results:**

Overall image quality for TSE_DL_ was rated to be excellent (median 5, IQR 4–5), significantly higher compared to TSE_S_ (median 5, IQR 4 – 5, *p* < 0.05), showing significantly lower extents of noise and improved sharpness (*p* < 0.001). Inter- and intra-reader agreement was almost perfect (*κ* = 0.92–1.00) for the detection of internal derangement and substantial to almost perfect (*κ* = 0.58–0.98) for the assessment of cartilage defects. No difference was found concerning the detection of bone marrow edema and fractures. The diagnostic confidence of TSE_DL_ was rated to be comparable to that of TSE_S_ (median 5, IQR 5–5, *p* > 0.05). Time of acquisition could be reduced to 6:11 min using TSE_DL_ compared to 11:56 min for a protocol using TSE_S_.

**Conclusion:**

TSE_DL_ of the knee is clinically feasible, showing excellent image quality and equivalent diagnostic performance compared to TSE_S_, reducing the acquisition time about 50%.

**Key Points:**

*• Deep-learning reconstructed TSE imaging is able to almost halve the acquisition time of a three-plane knee MRI with proton density and T1-weighted images, from 11:56 min to 6:11 min at 3 T.*

*• Deep-learning reconstructed TSE imaging of the knee provided significant improvement of noise levels (p < 0.001), providing higher image quality (p < 0.05) compared to conventional TSE imaging.*

*• Deep-learning reconstructed TSE imaging of the knee had similar diagnostic performance for internal derangement of the knee compared to standard TSE.*

**Supplementary Information:**

The online version contains supplementary material available at 10.1007/s00330-022-08753-z.

## Introduction

Magnetic resonance imaging (MRI) of the knee is among the most commonly performed MRI examinations and requires about 15 min of acquisition time. Reference standards for knee MRI are proton density (PD)– and T1–weighted turbo spin-echo (TSE) sequences due to the excellent tissue contrast and high in-plane spatial resolution with good assessment of meniscal, ligamentous, and cartilaginous injuries [1–3].

Due to their anisotropic voxel size, two-dimensional (2D)–TSE sequences require the acquisition of different image planes separately, which is time consuming [4, 5]. One approach to accelerate MRI of the knee is three-dimensional (3D)–TSE techniques, generating isotropic data sets of higher spatial resolution to create virtually any image plane from a single parental data set [4, 6]. Although technical developments can provide accelerated imaging, mainly based on parallel imaging (PI) acceleration, the acquisition time of a high-quality isotropic data set with 3D–TSE requires still around 5 to 10 min [4, 7]. Besides, with increasing acceleration in PI, the signal-to-noise ratio (SNR) decreases rapidly, while residual artifacts are generally increased which limits the achievable speed [8].

Another innovative technique, which is commonly used to accelerate MRI, is compressed sensing (CS), in which only a reduced set of data points is required. SNR is preserved better than by PI only, but CS tends to oversimplify image content, resulting in residual blurring and loss of realistic image textures.

The latest promising approaches to overcome this drawback are deep-learning (DL) algorithms. These feature trainable components in contrast to a priori assumptions on sparsity and promise higher acceleration factors while simultaneously increasing SNR and preserving high image quality [9, 10]. With regard to MRI of the knee, a recently published study using retrospectively undersampled data showed that DL images perform interchangeably with standard clinical images for the detection of internal derangement of the knee [8]. Furthermore, retrospectively undersampled, DL–accelerated images were rated with higher image quality than standard imaging and allowed an acceleration of the standard images [8]. There have been other technical developments of the DL reconstruction recently, but so far, there has been no prospective clinical study at both 1.5 and 3 T.

Our hypothesis was that TSE_DL_ can produce similar image quality that is comparable to clinically used segmented sequences while significantly reducing the acquisition time. Therefore, we implemented TSE_DL_ at 1.5 and 3 T in a prospective study to assess diagnostic performance compared to standard imaging sequences in routine clinical practice.

## Materials and methods

### Study design

Institutional review board approval and written informed consent from all participants were obtained for this prospective, single-center study. All study procedures were conducted in accordance with the ethical standards as laid down in the 1964 Declaration of Helsinki and its later amendments.

**Fig. 1:**
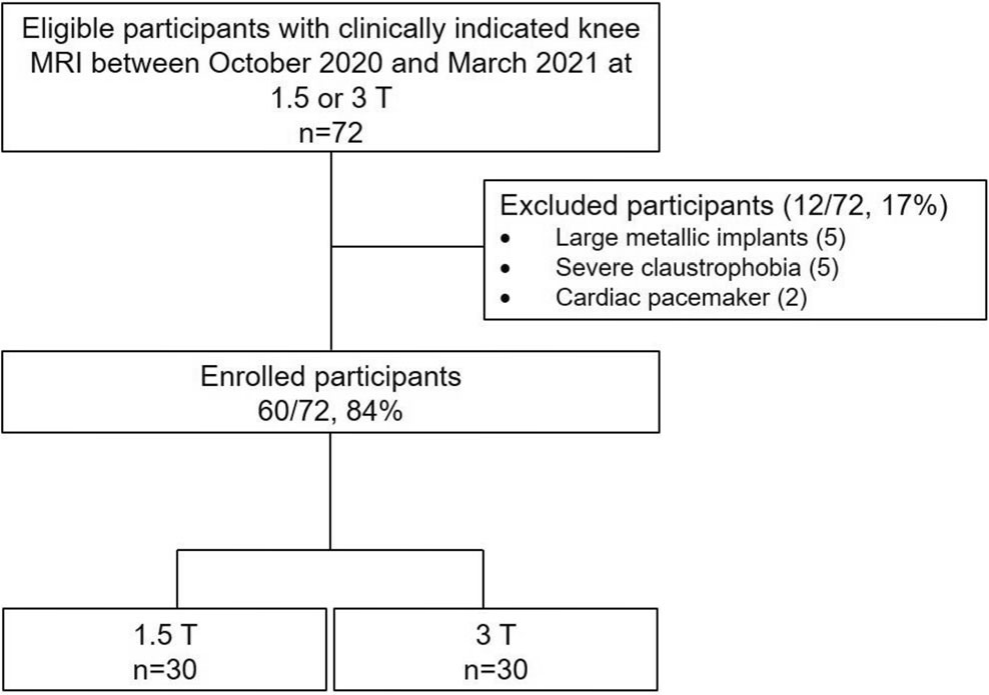
Flowchart of study participants

The power calculation for our sample size estimation revealed a sample size of 60 subjects using a test for agreement between two raters (kappa statistics) with 80% power to detect a true kappa value of 0.80 in a test with two categories with frequencies equal to 0.35 and 0.65 based on a significance level of 0.05 [11]. Study recruitment commenced consecutively from October 2020 to March 2021. Adult patients who underwent clinically indicated knee MRI were prospectively included. Exclusion criteria were general contraindications for MRI or incomplete study protocol. A final sample of 60 participants was included (see Fig. 1 and Table 1).

**Table 1:** Participants’ characteristics

Characteristics	Values
Total (male/female), *n*	60 (29/31)
Age, mean ± SD (range), y	Total: 44 ± 17 (18–85)
	Male: 46 ± 19 (18–85)
	Female: 42 ± 15 (19–78)
Indication of MRI, *n*	Pain, 25
	Suspected meniscal lesion, 16
	Trauma/distortion, 12
	Postoperative, 4
	Other or none, 10

*SD* standard deviation, *y* years, *n* number

### MRI system and acquisition parameters

All examinations were performed on clinical 1.5-T and 3-T scanners (MAGNETOM Skyra, MAGNETOM Prisma^fit^, MAGNETOM Vida, MAGNETOM Aera, and MAGNETOM Avanto; all Siemens Healthineers) with participants in supine position using clinical knee surface coils. All participants underwent our clinical standard knee MRI protocol including 2D-PD TSE_S_ with fat suppression in three planes (coronal, sagittal, and axial) and 2D-PD TSE_DL_ with fat suppression in three planes (coronal, sagittal, and axial), as well as 2D-T1-weighted TSE_S_ and 2D-T1-weighted TSE_DL_ in coronal orientation. Imaging parameters are displayed in Table 2.

**Table 2:** MRI acquisition parameters

	Sequence	FS	Orientation	TA	Acquired voxel size	Slices	FOV	TE	TR	FA	AV	C	PAT	TF
3T	PD TSE_S_	FS	Sagittal	3:11	0.67 × 0.47 × 3.0	30	150 × 150	44	3790	150	2	1	3	7
	PD TSE_DL_	FS	Sagittal	1:33	0.69 × 0.47 × 3.0	30	150 × 150	41	3580	150	1	1	3	7
	PD TSE_S_	FS	Coronal	3:11	0.67 × 0.47 × 3.0	30	150 × 150	44	3790	150	2	1	3	7
	PD TSE_DL_	FS	Coronal	1:33	0.69 × 0.47 × 3.0	30	150 × 150	41	3580	150	1	1	3	7
	PD TSE_S_	FS	Axial	3:11	0.67 × 0.47 × 3.0	30	150 × 150	44	3790	150	2	1	3	7
	PD TSE_DL_	FS	Axial	1:33	0.69 × 0.47 × 3.0	30	150 × 150	41	3580	150	1	1	3	7
	T1w TSE_S_		Coronal	2:23	0.42 × 0.33 × 3.0	30	150 × 150	10	566	150	1	1	2	3
	T1w TSE_DL_		Coronal	1:32	0.42 × 0.33 × 3.0	30	150 × 150	12	448	150	1	3	4	3
1.5T	PD TSE_S_	FS	Sagittal	3:12	0.67 × 0.47 × 3.0	30	150 × 150	42	3800	150	2	1	3	7
	PD TSE_DL_	FS	Sagittal	1:18	0.65 × 0.47 × 3.0	30	150 × 150	40	3630	150	1	1	4	7
	PD TSE_S_	FS	Coronal	3:12	0.67 × 0.47 × 3.0	30	150 × 150	42	3800	150	2	1	3	7
	PD TSE_DL_	FS	Coronal	1:18	0.65 × 0.47 × 3.0	30	150 × 150	40	3630	150	1	1	4	7
	PD TSE_S_	FS	Axial	1:56	0.67 × 0.47 × 3.0	30	150 × 150	16	3800	150	2	1	2	7
	PD TSE_DL_	FS	Axial	1:00	0.74 × 0.47 × 3.0	30	150 × 150	15	3420	150	1	1	4	7
	T1w TSE_S_		Coronal	2:13	0.42 × 0.33 × 3.0	30	150 × 150	9	527	150	1	2	2	3
	T1w TSE_DL_		Coronal	1:14	0.47 × 0.33 × 3.0	30	150 × 150	10	593	150	1	2	4	3

Acquired voxel size in mm^3^

*TA* time of acquisition, *FOV* field of view (mm^2^), *TE/TR* echo time/repetition time (ms); *FA* flip angle (degree), *AV* averages, *C* concatenations, *PAT* parallel acquisition technique, *TF* turbo factor, *TSE* turbo spin echo, *PD* proton density, *T1w* T1-weighted, *FS* fat saturation

### TSE with DL reconstruction

On the acquisition side, a conventional under-sampling pattern known from PI is used [10, 12], which provides the same performance when reconstructed with DL–based methods as incoherent sampling patterns favored by CS. The prototype image reconstruction comprises a fixed iterative reconstruction scheme or variational network [9, 10, 13]. For the image reconstruction, *k-space* data, bias-field correction and coil-sensitivity maps are inserted into the variational network. The fixed unrolled algorithm for accelerated MRI reconstruction consists of multiple cascades, each made up from a data consistency using a trainable Nesterov Momentum followed by a convolutional neural network (CNN)–based regularization [13].

The reconstruction was trained on prior volunteer acquisitions using conventional TSE protocols. About 10,000 slices were acquired on volunteers using various clinical 1.5-T and 3-T scanners (MAGNETOM scanners, Siemens Healthineers).

A detailed description of the used reconstruction is given in prior studies [13]. Besides this physics-based k-space to image reconstruction method, no other DL–based image–enhancement techniques such as super-resolution methods [14, 15] were employed in this study.

### Image evaluation

Corresponding TSE datasets have been separated for TSE_S_ and TSE_DL_, and each dataset was independently evaluated by radiologists with 3 to 9 years of experience in interpreting musculoskeletal MRI. The readers were blinded toward all participant information, reconstruction type, and clinical and radiological reports as well as each other’s assessments. Prior to the actual image analysis, each reader had received a training session to familiarize themselves with the Likert-scale classification. Image analysis was performed on a PACS workstation (GE Healthcare Centricity™ PACS RA1000). PD– and T1–weighted images were evaluated separately regarding overall image quality, artifacts, banding artifacts, sharpness, noise, diagnostic confidence, and subjective SNR using a 5-point Likert scale (1 = non-diagnostic; 2 = low image quality; 3 = moderate image quality; 4 = good image quality; 5 = excellent image quality). Reading scores were considered sufficient when reaching ≥ 3. Banding artifacts are characteristic artifacts produced by Cartesian DL reconstruction, particularly strong in low-SNR regions of the reconstructed image, appearing as a streaking pattern exactly aligned with the phase-encoding direction [16]. Furthermore, TSE_S_ and TSE_DL_ were evaluated regarding the image impression using a Likert scale ranging from 1 to 5 (1 = unrealistic; 5 = realistic).

Assessment of pathologies and internal derangement were conducted by the same three radiologists and included the evaluation of the medial and lateral menisci; medial and lateral collateral ligaments; anterior and posterior cruciate ligaments; and cartilage defects of the medial and lateral femur trochlea, the medial tibia plateau, the trochlear groove, and the retropatellar cartilage. Structural abnormalities were graded as 0 = normal, 1 = altered (degenerative, postoperative), and 2 = tear. Cartilage defects were classified using a modified version of the classification system of the International Cartilage Repair Society (ICRS). If more than one cartilage defect was present, only the dominant cartilage lesion was considered. Areas of bone marrow edema (femoral, patellar, tibial), as well as fractures and joint effusion, were evaluated being present or absent. If there were discrepancies between the readers, a consensus reading was enclosed to define false-positive and false-negative findings. All evaluated items of anatomic structures and pathologies are displayed in Table 3.

**Table 3:** Evaluated items of anatomic structures and pathologies of the knee

Item	Grade	Description/MRI finding
Meniscus	0	Normal
	1	Altered (degenerative or postsurgical changes)
	2	Tear (high signal intensity breaching the lower and/or upper meniscal surface)
Ligaments	0	Normal
	1	Altered (degenerative or postsurgical changes)
	2	Tear (discontinuity of at least 50% of the fibers)
Cartilage	0	Normal
	1	Nearly normal (superficial lesions: soft indentation and/or superficial fissures and cracks)
	2	Abnormal (lesions extending down to < 50% of cartilage depth)
	3a	Severely abnormal (cartilage defects extending down > 50% of cartilage depth)
	3b	Severely abnormal (cartilage defects extending down to calcified layer)
	3c	Severely abnormal (cartilage defects extending down to but not through the subchondral bone)
	4	Severely abnormal (penetration of the subchondral bone)
Bone marrow edema	0	Absent
	1	Present
Fracture	0	Absent
	1	Present
Joint effusion	0	Absent
	1	Present

### Statistical analysis

Statistical analyses were performed using SPSS version 26 (IBM Corp). Participants’ demographics and clinical characteristics were summarized by using descriptive statistics. Qualitative image analysis assessment was given as mean and median values with interquartile range (IQR). An exact paired-sample Wilcoxon signed-rank test was used to compare the sequences in terms of the image quality scores from each reader. A post hoc multinominal regression analysis (generalized linear model for ordinal variables) was computed for the impact of field strength, reader, and patient demographics. Significance was assumed at a level of *p* < 0.05.

Inter-reader agreement of the three readers was assessed by using Fleiss’ *κ* and intra-reader agreement by using weighted Cohen’s *κ*, both with 95% confidence intervals and interpreted as follows: 0.20 or less, poor agreement; 0.21–0.40, fair agreement; 0.41–0.60, moderate agreement; 0.61–0.80, substantial agreement; and greater than 0.80, almost perfect agreement.

## Results

Among 72 eligible participants, a final sample of 60 participants (84%, mean age 44 ± 17; range 18–85 (years); 29 males, 31 females) were prospectively included in this study. Thirty examinations were performed on 1.5 T and 30 examinations on 3 T regardless of diagnosis, current treatment, first examination, or follow-up (Table 1 and Fig. 1).

### Image quality

Inter-reader agreement was substantial to almost perfect with values between 0.61 and 0.85 (see Table 4). Because of the good inter-reader reliability, in the following, only the results of reader 1 are given. A summary of all qualitative image analyses and Fleiss’ *κ* are provided in Table 4.

**Table 4:** Image quality and inter-reader agreement of standard TSE (TSE_S_) and deep-learning-reconstructed TSE (TSE_DL_)

Sequence	Item	Reader 1			Reader 2			Reader 3			Fleiss’ *κ*	
		TSE_S_	TSE_DL_	*p* value	TSE_S_	TSE_DL_	*p* value	TSE_S_	TSE_DL_	*p* value	TSE_S_	TSE_DL_
PD TSE	Overall image quality	4.57 [5 (4–5)]	4.80 5 (5–5)	0.003	4.58 [5 (4–5)]	4.78 [5 (5–5)]	0.007	4.55 [5 (4 – 5)]	4.82 [5 (5–5)]	0.003	0.71	0.75
	Artifacts	4.82 [5 (5–5)]	4.75 [5 (5–5)]	0.38	4.78 [5 (5–5)]	4.75 [5 (5–5)]	0.61	4.80 [5 (5 – 5)]	4.65 [5 (4–5)]	0.10	0.70	0.69
	Banding artifacts	4.97 [5 (5–5)]	4.43 [5 (4–5)]	< 0.001	4.98 [5 (5–5)]	4.42 [4 (4–5)]	< 0.001	4.98 [5 (5 – 5)]	4.48 [5 (4–5)]	< 0.001	0.74	0.80
	Sharpness	4.65 [5 (4–5)]	4.88 [5 (5–5)]	< 0.001	4.57 [5 (4–5)]	4.88 [5 (5–5)]	< 0.001	4.55 [5 (4 – 5)]	4.88 [5 (5–5)]	< 0.001	0.68	0.70
	Noise	4.30 [4 (4–5)]	4.90 [5 (5–5)]	< 0.001	4.33 [4 (4–5)]	4.92 [5 (5–5)]	< 0.001	4.37 [4.5 (4 – 5)]	4.90 [5 (5–5)]	< 0.001	0.66	0.74
	Diagnostic confidence	4.90 [5 (5–5)]	4.93 [5 (5–5)]	0.59	4.87 [5 (5–5)]	4.92 [5 (5–5)]	0.32	4.82 5 (5 – 5)]	4.93 [5 (5–5)]	0.08	0.68	0.81
	Subjective SNR	4.53 [5 (4–5)]	4.88 [5 (5–5)]	< 0.001	4.53 [5 (4–5)]	4.87 [5 (5–5)]	< 0.001	4.60 [5 (4 – 5)]	4.85 [5 (5–5)]	0.003	0.69	0.78
T1 TSE	Overall image quality	4.90 [5 (5–5)]	4.97 [5 (5–5)]	0.046	4.88 [5 (5–5)]	4.97 [5 (5–5)]	0.03	4.88 [5 (5 – 5)]	4.98 [5 (5–5)]	0.03	0.77	0.79
	Artifacts	4.95 [5 (5–5)]	4.95 [5 (5–5)]	< 0.99	4.95 [5 (5–5)]	4.95 [5 (5–5)]	< 0.99	4.93 [5 (5 – 5)]	4.98 [5 (5–5)]	0.08	0.79	0.70
	Banding artifacts	4.97 [5 (5–5)]	4.92 [5 (5–5)]	0.10	4.95 [5 (5–5]	4.93 [5 (5–5)]	0.48	4.97 [5 (5 – 5)]	4.95 [5 (5–5)]	0.71	0.66	0.64
	Sharpness	4.92 [5 (5–5)]	4.93 [5 (5–5)]	0.74	4.90 [5 (5– 5)]	4.93 [5 (5–5)]	0.53	4.93 [5 (5 – 5)]	4.95 [5 (5–5)]	0.71	0.78	0.61
	Noise	4.78 [5 (5–5)]	4.97 [5 (5–5)]	0.002	4.75 [5 (4.25–5)]	4.98 [5 (5–5)]	< 0.001	4.87 [5 (5 – 5)]	4.98 [5 (5–5)]	0.02	0.69	0.74
	Diagnostic confidence	4.97 [5 (5–5)]	4.97 [5 (5–5)]	< 0.99	4.97 [5 (5–5)]	4.98 [5 (5–5)]	0.32	4.95 [5 (5 – 5)]	4.98 [5 (5–5)]	0.16	0.85	0.74
	Subjective SNR	4.92 [5 (5–5)]	4.97 [5 (5–5)]	0.18	4.88 [5 (5–5)]	4.95 [5 (5–5)]	0.10	4.95 [5 (5 – 5)]	4.97 [5 (5–5)]	0.56	0.64	0.70
Image impression		4.83 [5 (5–5)]	4.45 [4 (4–5)]	< 0.001	4.82 [5 (5–5)]	4.43 [4 (4–5)]	< 0.001	4.82 [5 (5 – 5)]	4.40 [4 (4–5)]	< 0.001	0.88	0.83

The results are reported as mean [median (interquartile range)]

*SNR* signal-to-noise ratio, *Fleiss’ κ* inter-reader agreement between the three readers

With regard to the PD sequences, overall image quality was rated highest for TSE_DL_ (median 5, IQR 5 – 5), significantly higher compared to TSE_S_ (median 5, IQR 5 – 5, *p* = 0.003). Sharpness, noise, and subjective SNR were also rated to be significantly higher in TSE_DL_ (median 5, IQR 5–5) compared to TSE_S_ (median 5, IQR 4–5, *p* < 0.001). The extent of artifacts was rated to be similar between TSE_DL_ and TSE_S_ (median 5, IQR 5–5, *p* > 0.05), although TSE_DL_ was rated to show significantly more banding artifacts (median 5, IQR 4–5) compared to TSE_S_ (median 5, IQR 5 – 5, *p* = 0.003). Nonetheless, no difference was found with reference to the diagnostic confidence of both sequences (median 5, IQR 5 – 5, *p* > 0.05).

Concerning the T1-weighted sequences, overall image quality was rated to be significantly higher in TSE_DL_ (median 5, IQR 5 – 5) compared to TSE_S_ (median 5, IQR 5 – 5, *p* = 0.046). Noise was evaluated significantly superior in TSE_DL_ (median 5, IQR 5 – 5) compared to TSE_S_ (median 5, IQR 5 – 5, *p* = 0.002). There was no significant difference regarding artifacts, banding artifacts, sharpness, diagnostic confidence, and subjective SNR between TSE_DL_ and TSE_S_ (median 5, IQR 5 – 5, *p* > 0.05).

For further illustration, raw data of a patient examined at 1.5 and of a patient examined at 3 T were exported and exemplary SNR maps were determined offline using a pseudo-replica method. Furthermore, the raw data of the TSE_DL_ acquisition was reconstructed using the DL technique and a conventional generalized autocalibrating partially parallel acquisition (GRAPPA) reconstruction to illustrate the differences between the reconstruction techniques. Note that noise is highest in images acquired at 1.5 T and reconstructed with the GRAPPA reconstruction; see Figs. 7, 8 and 9.

A post hoc multinomial regression analysis via a generalized linear model for ordinal variables was utilized to investigate whether “field strength” (1.5 T/3 T), patients’ demographics (sex and age), and “reader” (readers 1–3) could predict how noise and banding artifacts were rated for each reconstruction type (TSE_S_/TSE_DL_).

For noise in TSE_S_, the factor “field strength” was found to contribute to the model (*p* < 0.001), whereas the factor “reader” was not a significant contributor to the model (*p* > 0.05). For each deduction of noise by 1-point decrease on the Likert scale, the likelihood of the image being scanned on a 1.5-T scanner was almost 19-fold (odds ratio 18.5, 95% CI [8.8–39]).

For noise in TSE_DL_, the factor “field strength” was not a significant contributor to the model (> 0.05).

For banding artifacts in TSE_DL_, the factor “field strength” was found to contribute to the model (*p* < 0.001), whereas the factor “reader” was not a significant contributor to the model (*p* > 0.05). For each improvement of noise by 1-point increase on the Likert scale, the likelihood of the image being scanned on a 3-T scanner was almost 11-fold (odds ratio 10.9, 95% CI [5.4–22.1]). For banding artifacts in TSE_S_, the factor “field strength” was not a significant contributor to the model (> 0.05).

For image quality in TSE_DL_ and TSE_S_, the patient demographic factors “sex” and “age” were not significant contributors to the model (> 0.05).

### Visibility of anatomic structures and internal derangement

Concerning the detection of degeneration or tears of the menisci and ligaments, inter- and intra-reader agreement was almost perfect with *κ* values between 0.92 and 1.00. There was no clinically relevant difference concerning the detection of structural abnormalities between TSE_S_ and TSE_DL_. Regarding the detection and evaluation of cartilage defects, inter- and intra-reader agreement was substantial to almost perfect with *κ* values between 0.58 and 0.98. No difference was found between the readers and the two sequences TSE_S_ and TSE_DL_ with regard to the detection of femoral, tibial, and patellar bone marrow edema, as well as regarding the detection of fractures. A total of four fractures were detected by all readers in both sequences. Inter- and intra-reader agreement was almost perfect with *κ* values between 0.89 and 0.97 for the presence of joint effusion.

**Fig. 2:**
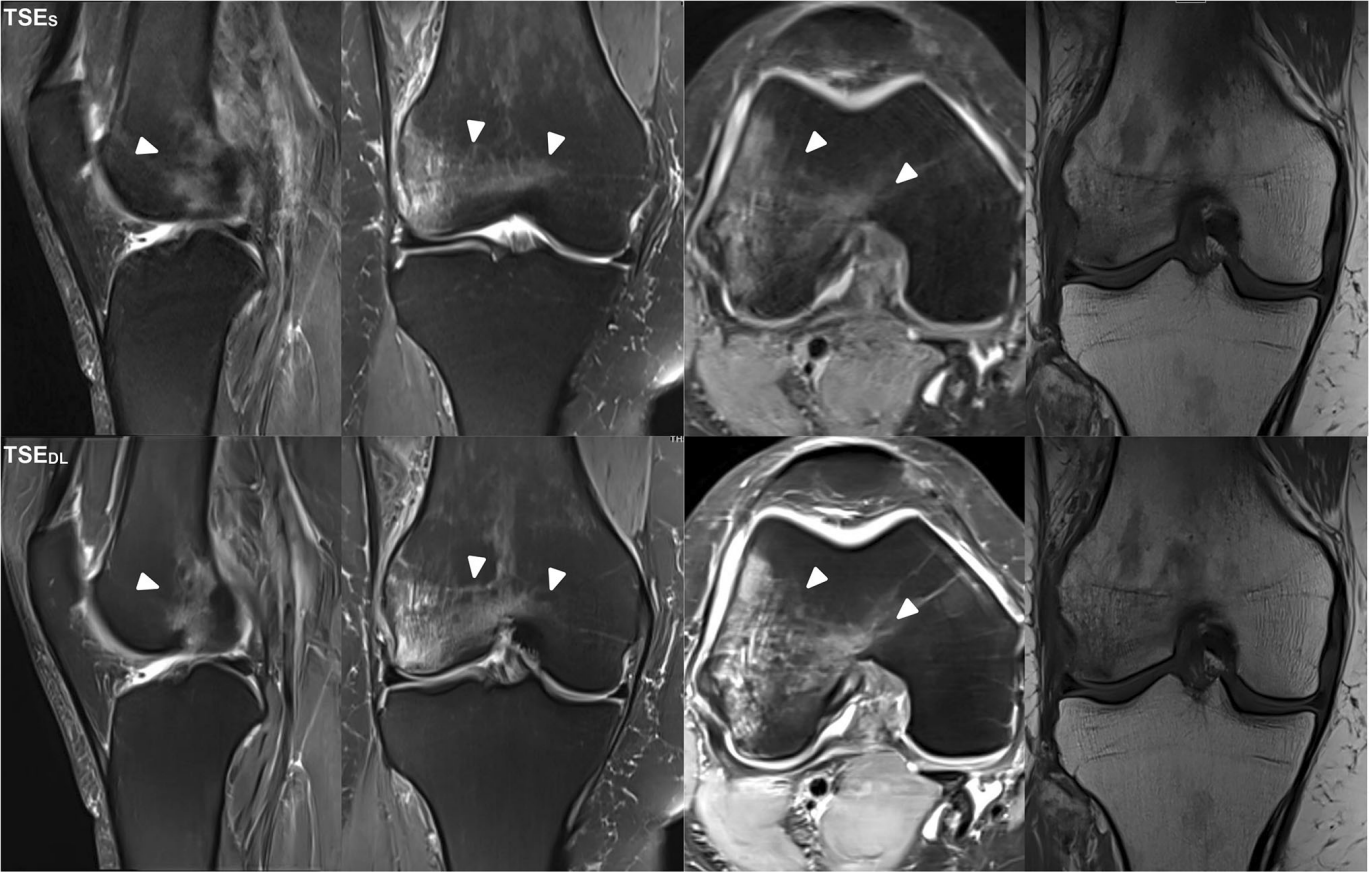
Image example of a standard and deep-learning-reconstructed PD TSE imaging of the knee at 3 T. This is an example of a comprehensive knee MRI at 3 T of a 46-year-old patient with pain in the medial side of the right knee after trauma. PD– and T1–weighted TSE_S_ (upper row) and TSE_DL_ (lower row) in different orientations are compared. TSE_DL_ provides higher image quality with lower extents of noise and improved sharpness of the anatomic structures. Note that bone marrow edema (white arrowheads) of the femoral condyle is clearly definable in both TSE_S_ and TSE_DL_

**Fig. 3:**
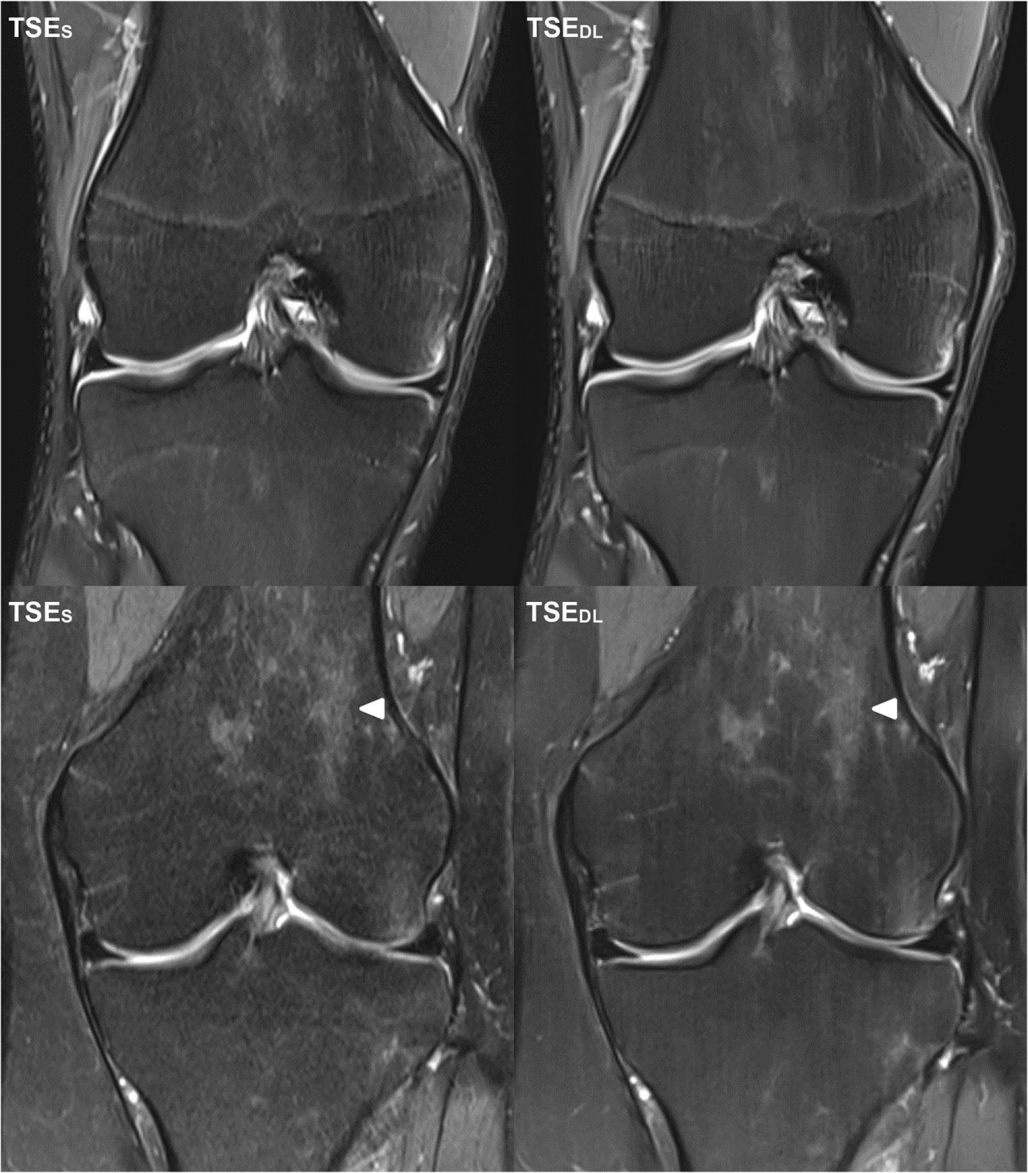
Image examples of a standard and deep-learning-reconstructed PD TSE imaging of the knee at 3 T and 1.5 T. The upper-row images are examples of knee MRI at 3 T in coronal orientation of an 18-year-old professional athlete with pain in the area of the patella of both knees. After a break from training, the complaints had improved. The lower-row images are examples of knee MRI at 1.5 T in coronal orientation (lower row, PD TSE_S_ left and PD TSE_DL_ right) of a 30-year-old patient after knee distortion. Comparing PD TSE_S_ (left) and PD TSE_DL_ (right), in PD TSE_DL_, the difference in the extents of noise at 3 T (upper row) is less present than in images acquired at 1.5 T (lower row). Unfortunately, TSE_DL_ images at 1.5 (lower row, right) show characteristic banding artifacts (white arrowheads), known as streaking, which are not present in TSE_S_

**Fig. 4:**
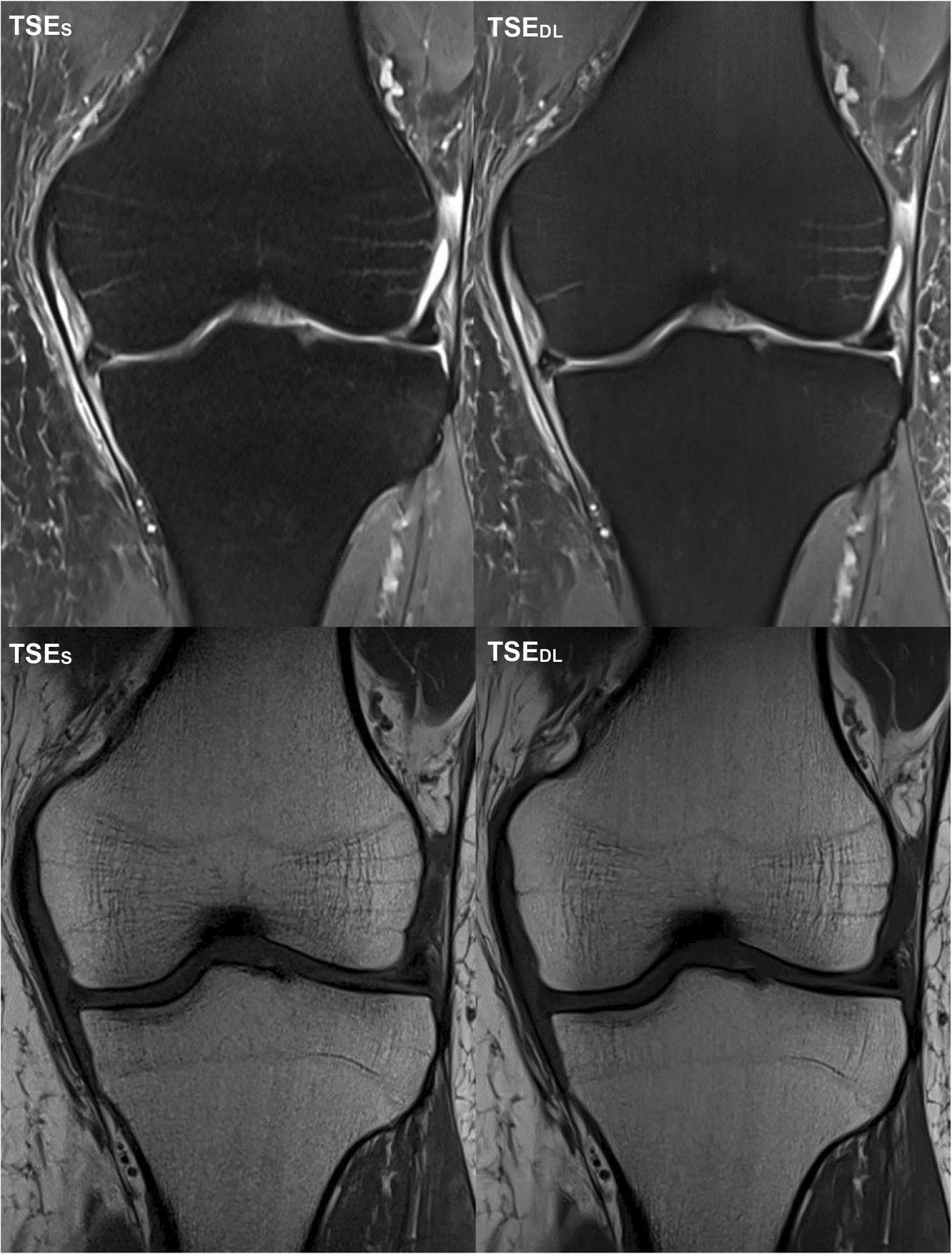
Image example of a standard and deep-learning-reconstructed PD– and T1–weighted TSE imaging of the knee at 1.5 T. This is an example of a knee MRI at 1.5 T in coronal orientation of a 59-year-old patient after partial resection of the medial meniscus. PD TSE_S_ (upper row, left) and PD TSE_DL_ (upper row, right) and T1w TSE_S_ (lower row, left) and PD TSE_DL_ (lower row, right). Comparing PD TSE (upper row) and T1w TSE (lower row), the effect of the noise reduction is more present in PD TSE_DL_ compared to PD TSE_S_ than comparing T1w TSE_DL_ compared to T1w TSE_S_

**Fig. 5:**
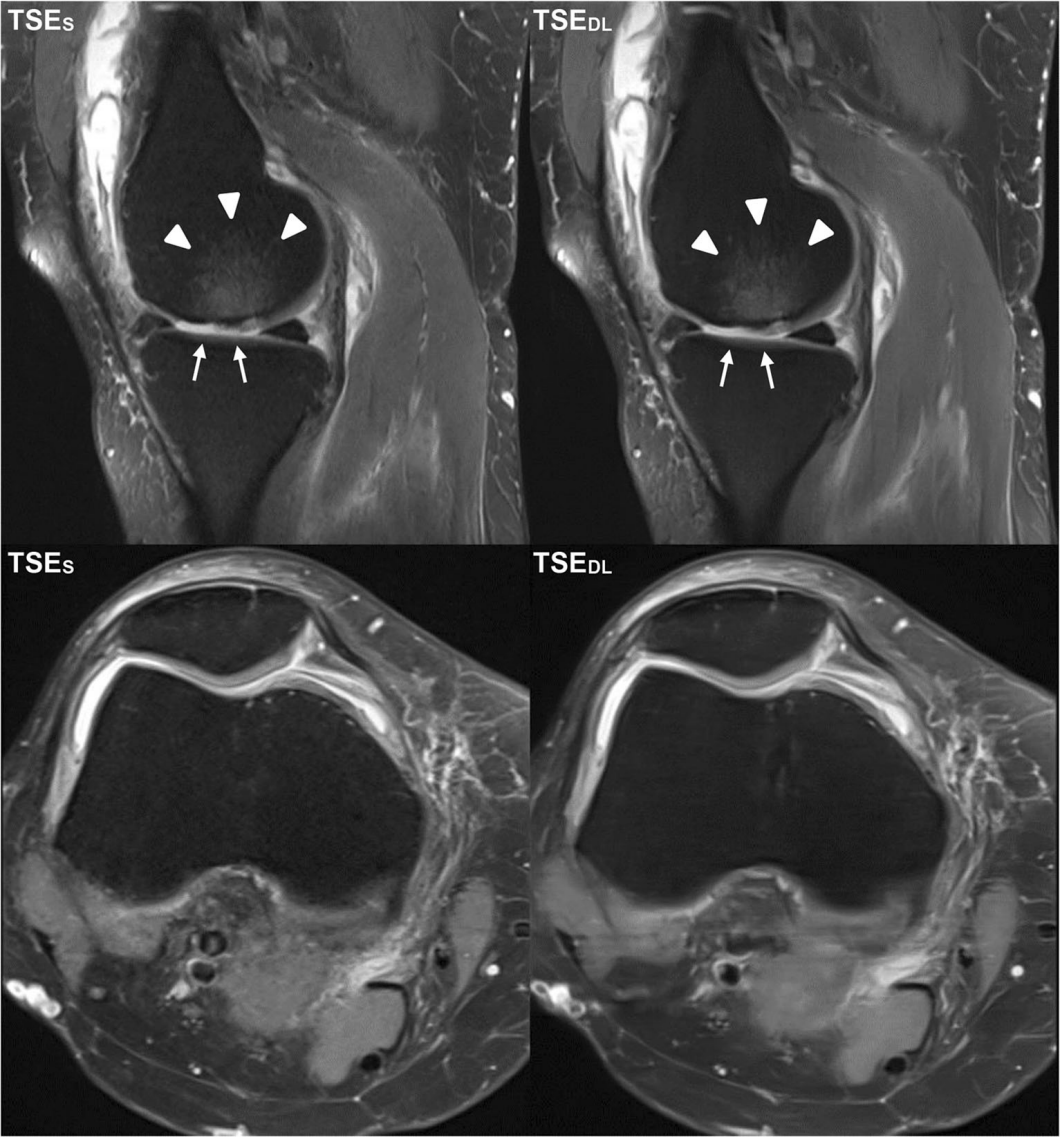
Image example of a standard and deep-learning-reconstructed TSE imaging of the knee at 1.5 T. This is an example of a knee MRI at 1.5 T in sagittal and axial orientation of a 52-year-old patient with pain in the medial side of the right knee. In the sagittal images, the cartilage defect (ICRS grade 4; white arrows) of the medial femoral condyle with adjacent bone marrow edema (white arrowheads) is visible in both TSE_S_ (left) and TSE_DL_ (right). Comparing TSE_S_ (left) and TSE_DL_ (right) especially in the axial orientation, TSE_DL_ shows characteristic banding artifacts of deep-learning-accelerated images when acquired at 1.5 T

**Fig. 6:**
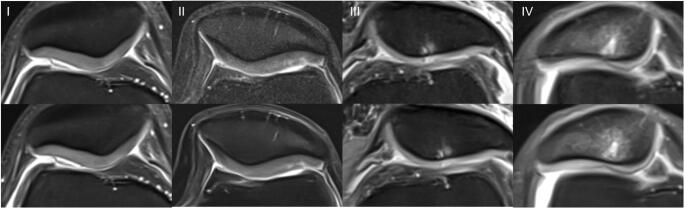
Image example of a standard and deep-learning-reconstructed TSE imaging of the knee. This is an example of a knee MRI in axial orientation comparing the cartilage defects (ICRS grades 1 to 4, from left to right) of the retropatellar cartilage in both TSE_S_ (upper row) and TSE_DL_ (lower row). All cartilage defects are definable in both sequences TSE_S_ and TSE_DL_

Intra- and inter-reader agreement of detected pathologies is summarized in Table 5. An overview of all detected pathologies is displayed as supplemental material (Table 6). Image examples of TSE_S_ and TSE_DL_ are provided in Figs. 2, 3, 4, 5 and 6.

**Table 5:** Intra- and inter-reader agreement of detected pathologies in standard TSE (TSE_S_) and deep-learning-reconstructed TSE imaging (TSE_DL_)

Item	Location	Cohen’s *κ*			Fleiss’ *κ*	
		R1	R2	R3	TSE_S_	TSE_DL_
Degeneration/tear	Medial meniscus	1.00	1.00	0.98	0.97	0.95
	Lateral meniscus	1.00	1.00	1.00	0.92	0.92
	MCL	1.00	1.00	1.00	0.96	0.96
	LCL	1.00	1.00	1.00	1.00	1.00
	ACL	0.94	1.00	1.00	0.92	0.97
	PCL	1.00	1.00	1.00	1.00	1.00
Cartilage defects	Total	0.86	0.85	0.95	0.84	0.72
	MFC	0.86	0.82	0.83	0.79	0.66
	LFC	0.64	0.58	0.91	0.79	0.62
	MTP	0.88	0.84	0.86	0.76	0.64
	LTP	0.76	0.83	0.87	0.88	0.89
	Trochlear groove	0.92	0.87	0.96	0.78	0.76
	Retropatellar	0.85	0.80	0.96	0.77	0.64
Bone marrow edema	Total	1.00	1.00	1.00	1.00	1.00
	Femoral	1.00	1.00	1.00	1.00	1.00
	Tibial	1.00	1.00	1.00	1.00	1.00
	Patellar	1.00	1.00	1.00	1.00	1.00
Fracture		1.00	1.00	1.00	1.00	1.00
Joint effusion		0.89	0.89	0.89	0.97	0.93

*MCL* medial collateral ligament, *LCL* lateral collateral ligament, *ACL* anterior cruciate ligament, *PCL* posterior cruciate ligament, *MFC* medial femoral condyle, *LFC* lateral femoral condyle, *MTP* medial tibial plateau, *LTP* lateral tibial plateau, *Cohen’s κ* intra-reader agreement between TSE_S_ and TSE_DL_, *Fleiss’ κ* inter-reader agreement

## Discussion

In this study, we investigated the feasibility and performance of a deep-learning-based reconstruction for 2D–TSE sequences (TSE_DL_) compared to standard 2D-TSE sequences concerning overall image quality items and the diagnosis of internal derangement of the knee at 1.5 T and 3 T. TSE_DL_ enables a robust and reliable acquisition of images in clinical routine practice, providing even higher overall image quality and equal diagnostic performance compared to TSE_S_ in a short acquisition time.

The current clinical standard for MRI examinations of the knee is a multi-plane 2D-TSE sequence, which is, due to its multiple planes and contrasts, time consuming, with an acquisition time of about 15 min. Several approaches have been made to accelerate knee imaging, especially promising 3D sequences such as 3D-TSE or 3D-SPACE [4, 7, 17] with the ability to create any imaging plane and slice thickness from a single volume. Regardless, the inverse relationship between acquisition time and image quality leads to relatively long acquisition times of about 10 min for small voxel sizes of (0.5 mm)^3^ [4, 7]. Small voxel sizes are needed to ensure the visibility of fine anatomic details and interplanar uniformity of reconstructions. Although several studies indicate the equality or even superiority of 3D sequences [7, 17–19], this technique has not yet been widely adopted in clinical practice and most study protocols consisted exclusively of PD-weighted images [7].

**Fig. 7:**
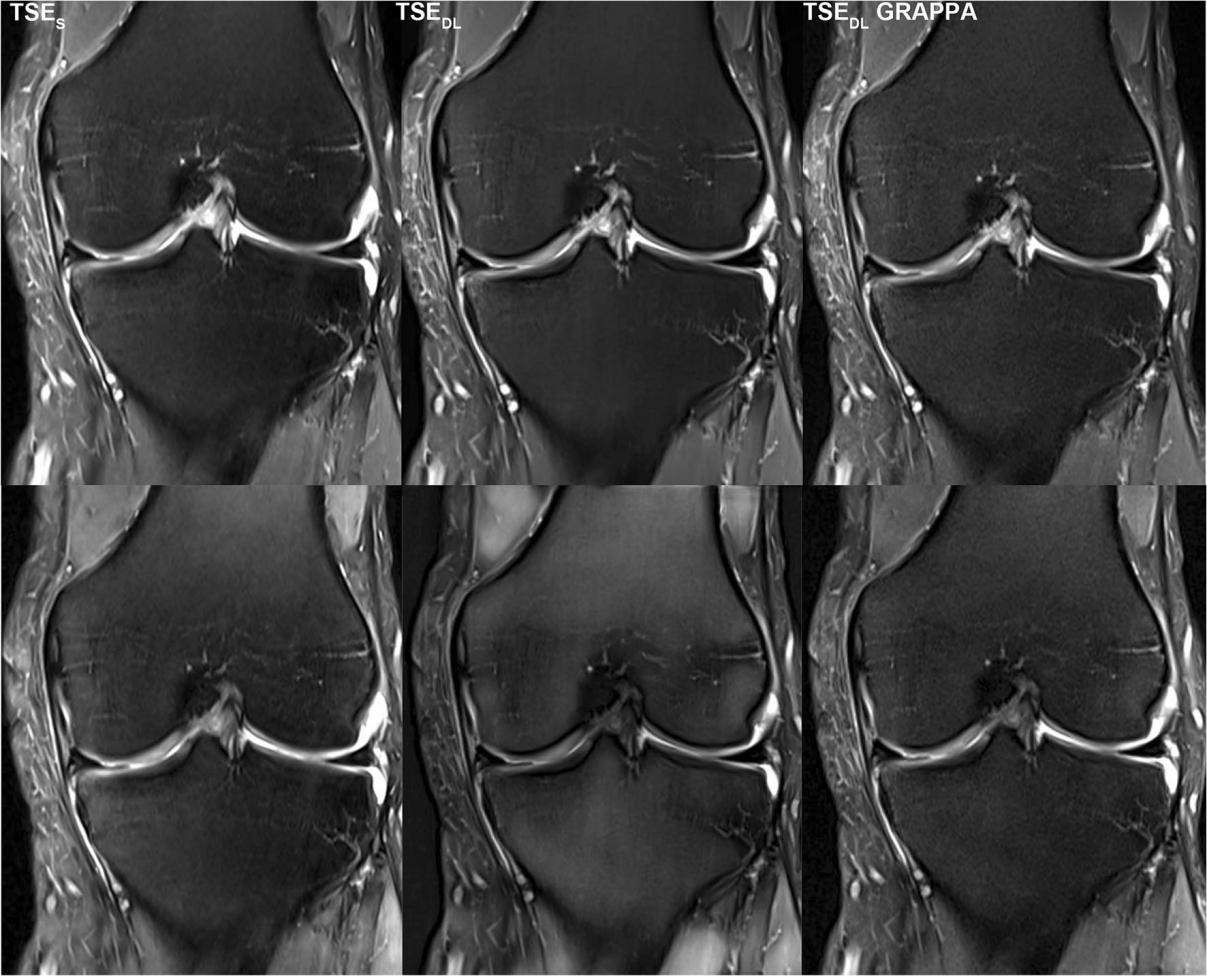
Comparison of different reconstruction techniques and SNR for standard and deep-learning PD TSE imaging of the knee at 3 T. Exemplary visualization of different reconstruction techniques (upper row) and signal-to-noise ratio (SNR) as SNR maps (lower row) of PD-weighted TSE in coronal orientation of the knee acquired at 3 T. On the left, TSE_S_ dataset reconstructed with a standard GRAPPA reconstruction. In the middle, TSE_DL_ dataset reconstructed with the DL technique and, on the right, TSE_DL_ dataset reconstructed with a standard GRAPPA reconstruction. Compared to the TSE_S_ (upper row, left), the TSE_DL_ reconstructed with GRAPPA (upper row, right) shows higher noise levels and a decrease of SNR (lower row, left and right). The TSE_DL_ reconstructed with the DL technique (upper row, middle) shows lower noise levels and an increase of SNR compared to both TSE_S_ and TSE_DL_ reconstructed with GRAPPA (lower row)

**Fig. 8:**
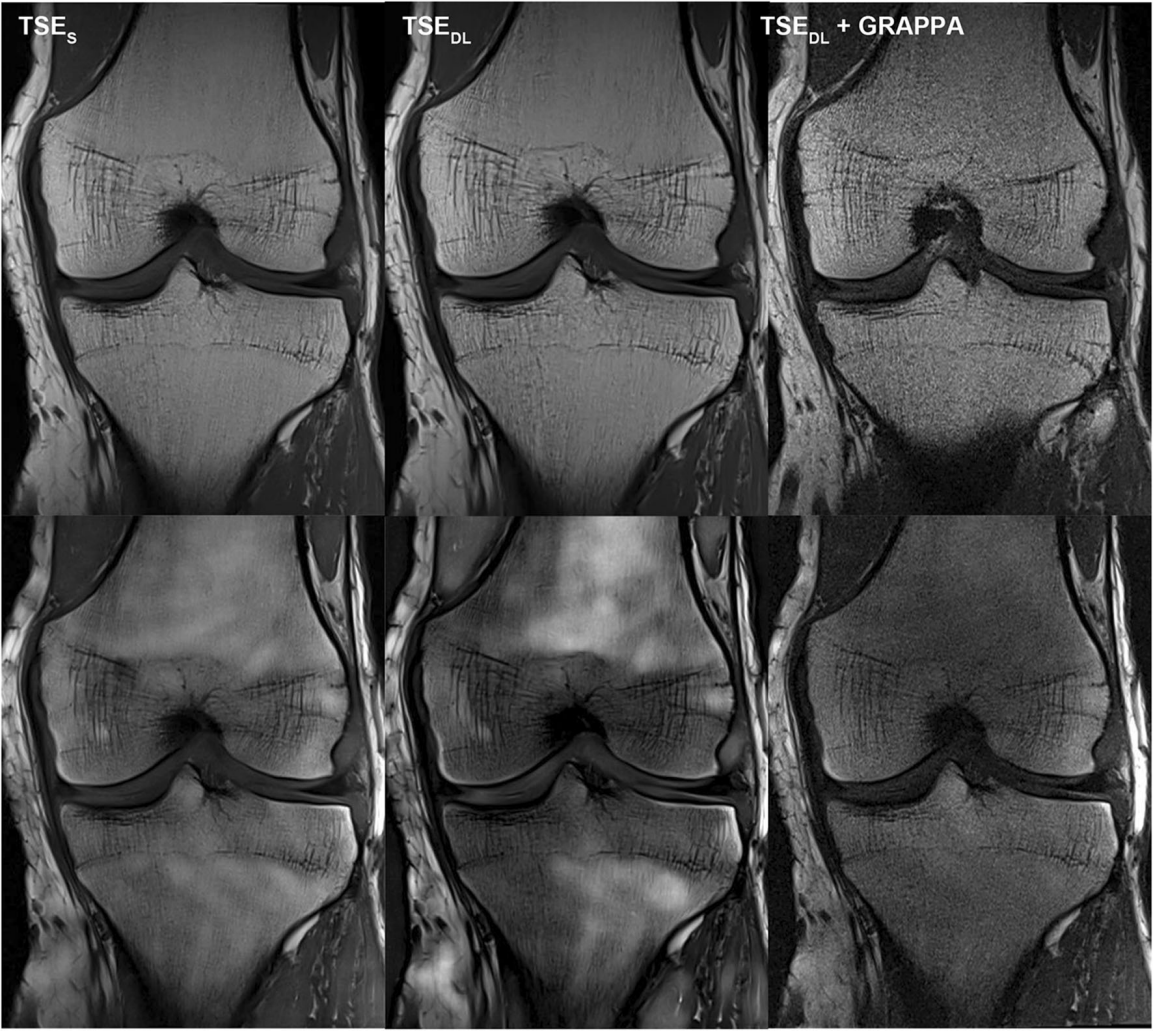
Comparison of different reconstruction techniques and SNR for standard and deep-learning T1 TSE imaging of the knee at 3 T. Exemplary visualization of different reconstruction techniques (upper row) and signal-to-noise ratio (SNR) as SNR maps (lower row) of T1-weighted TSE in coronal orientation of the knee acquired at 3 T. On the left, TSE_S_ dataset reconstructed with a standard GRAPPA reconstruction. In the middle, TSE_DL_ dataset reconstructed with the DL technique and, on the right, TSE_DL_ dataset reconstructed with a standard GRAPPA reconstruction. Compared to the TSE_S_ (upper row, left), the TSE_DL_ reconstructed with GRAPPA (upper row, right) shows higher noise levels and a decrease of SNR (lower row, left and right). The TSE_DL_ reconstructed with the DL technique (lower row, middle) shows lower noise levels and an increase of SNR compared to TSE_DL_ reconstructed with GRAPPA. TSE_S_ (left) and TSE_DL_ reconstructed with the DL technique (middle) are comparable concerning the noise levels and SNR

**Fig. 9:**
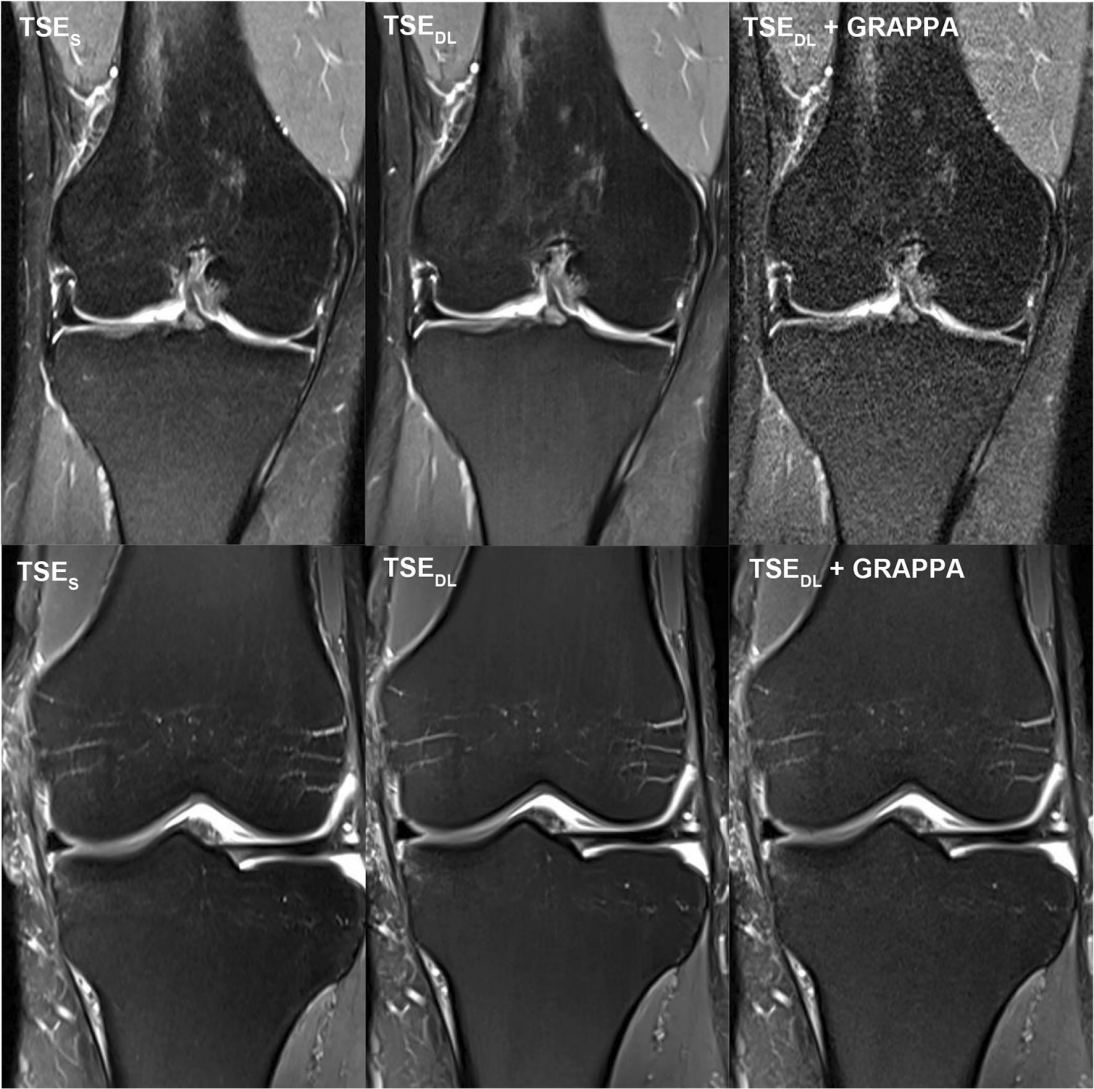
Comparison of different reconstruction techniques for standard and deep-learning PD TSE imaging of the knee at 1.5 and 3 T. Exemplary visualization of different reconstruction techniques at 1.5 T (upper row) and 3 T (lower row) of PD–weighted TSE in coronal orientation of the knee. On the left, TSE_S_ dataset reconstructed with a standard GRAPPA reconstruction. In the middle, TSE_DL_ dataset reconstructed with the DL technique and, on the right, TSE_DL_ dataset reconstructed with a standard GRAPPA reconstruction. Comparing the TSE_DL_ reconstructed with GRAPPA at 1.5 T (upper row, right) and 3 T, the image acquired at 3 T (lower row, right) shows less noise levels and the effects of the DL reconstruction are minor when images are acquired at 3 T

For the current standard 2D-TSE imaging of the knee, other acceleration techniques have been used, such as PI, CS, and simultaneous multi-slice [20–24]. Diagnostic equivalence can be obtained when using acceleration factors up to twofold. However, PI and simultaneous multi-slice may suffer from reduced SNR, noise enhancement, aliasing, and reconstruction artifacts, especially if higher acceleration factors are used [25, 26]. The immense potential of AI-based reconstruction techniques, such as deep learning, to accelerate MRI while maintaining or even improving the image quality, had been shown in several studies [8, 27–31]. According to these, in our study, TSE_DL_ enabled an improvement of the overall image quality and significantly reduced the extent of noise, especially for images acquired at 1.5 T. The acquisition time of a knee MRI can be reduced to 6:11 min using TSE_DL_ compared to 11:56 min for our standard protocol using TSE_S_. Even though the extent of general artifacts showed no difference between TSE_S_ and TSE_DL_, banding artifacts in images acquired at 1.5 T were present, which have been observed with multiple, different deep-learning reconstruction techniques [16]. They have been correlated to the Cartesian sampling scheme with integrated reference scans and are particularly strong in low signal-to-noise regions of the reconstructed image. As such, images acquired at 1.5 T and image contrasts with fat suppression are known to be more prone to banding artifacts (Figs. 7, 8 and 9). Coincidently, our PD protocols employed spectral fat suppression and therefore were more affected by banding artifacts. Recent approaches have shown promising results to reduce such banding artifacts [16]. However, although banding artifacts are present in TSE_DL_ and need to be reduced in further developments of the used network, they do not affect the diagnostic confidence of TSE_DL_.

Concerning the detection of internal derangement, there was no substantial difference between the TSE_S_ and TSE_DL_ sequences. Although intra- and inter-reader agreement for the presence of cartilage defects showed lower *κ* values, it would not have led to any change in therapy of the participants, and can be explained by the subjective reading, what is already described in literature [32].

With regard to the acquisition time of the MRI, in addition to the acceleration of the data acquisition, there is also another advantage compared to previously used acceleration techniques such as CS: Up to now, acceleration techniques suffered from long post-processing times and the need of high computational resources [33, 34]. The deep-learning approach stands out, due to the fact that most of the computational work has been done in advance during training of the network; thus, the reconstruction time of deep-learning-based sequences is very low.

Our findings should be interpreted within the context of the study’s limitations. First, while all readers were blinded to the shown sequences, the characteristic differences in the appearance allowed readers to recognize the reconstruction technique. Therefore, personal preferences may have influenced the study results. Second, in this study, just one network was used to reconstruct the undersampled image data, and this network was trained on various anatomic regions. Further improvements of the used first network have already been done and should be evaluated in further studies, especially with regard to the extent of banding artifacts at images of 1.5-T scanners. Third, all examinations were performed on MRI scanners produced by a single vendor. Further studies on multiple-vendor scanners are needed evaluating the performance of this network also with regard to other anatomic regions to entirely assess the generalizability of this technique.

In conclusion, our study indicates that TSE_DL_ is clinically feasible, providing even better image quality in a shorter acquisition time. Dependent on its ability to accurately reconstruct meniscus and ligament tears, TSE_DL_ yields comparable diagnostic performance for internal knee derangement to standard TSE.

## Supplementary Information

ESM 1 (DOCX 22 kb)

## Abbreviations

CS: Compressed sensing
DL: Deep learning
IQ: Image quality
IQR: Interquartile range
MRI: Magnetic resonance imaging
PI: Parallel imaging
PD: Proton density
SNR: Signal-to-noise ratio
S: Standard
3D: Three-dimensional
TSE: Turbo spin-echo
2D: Two-dimensional

## Declarations

### Guarantor

The scientific guarantor of this publication is Prof. Dr. med. Ahmed E. Othman.

### Statistics and biometry

No complex statistical methods were necessary for this paper.

### Methodology

• prospective

• observational

• performed at one institution
