# Supplementary material for: Feasibility of an accelerated 2D-multi-contrast knee MRI protocol using deep-learning image reconstruction: a prospective intraindividual comparison with a standard MRI protocol
Source: Eur Radiol. 2022 Apr 7;32(9):6215–29. doi: 10.1007/s00330-022-08753-z (PMC9381615; doi:10.1007/s00330-022-08753-z)
Supplement: Supplementary file 1 — (DOCX 22 kb) [file 330_2022_8753_MOESM1_ESM.docx]

**Supplemental Material:**

Table 6: Number of detected pathologies in standard TSE (TSE_S_) and deep-learning-reconstructed TSE imaging (TSE_DL_)

| **Item** | **Location** | **TSE_S_** | | | | | | | | | | | | | | | | | | | | | | | | | **TSE_DL_** | | | | | | | | | | | | | | | | | | | | | | | | | | | |  |
| --- | --- | --- | --- | --- | --- | --- | --- | --- | --- | --- | --- | --- | --- | --- | --- | --- | --- | --- | --- | --- | --- | --- | --- | --- | --- | --- | --- | --- | --- | --- | --- | --- | --- | --- | --- | --- | --- | --- | --- | --- | --- | --- | --- | --- | --- | --- | --- | --- | --- | --- | --- | --- | --- | --- | --- |
|  | | **0 = normal** | | | | | | | | | **1 = altered** | | | | | | | | **2 = tear** | | | | | | | | **0 = normal** | | | | | | | | **1 = altered** | | | | | | | | **2 = tear** | | | | | | | | | | | |  |
| Degeneration/tear |  | Reader 1-3 | | | | | | | | | Reader 1-3 | | | | | | | | Reader 1-3 | | | | | | | | Reader 1-3 | | | | | | | | Reader 1-3 | | | | | | | | Reader 1-3 | | | | | | | | | | | |  |
|  | Medial meniscus | 27 | | 27 | | | | 28 | | | 11 | | 11 | | | | 11 | | 22 | | 22 | | | | 21 | | 27 | | 27 | | | | 29 | | 11 | | 11 | | | | 10 | | 22 | | | 22 | | | | | 21 | | |  |  |
|  | Lateral meniscus | 51 | | 51 | | | | 50 | | | 4 | | 4 | | | | 6 | | 5 | | 5 | | | | 4 | | 51 | | 51 | | | | 50 | | 4 | | 4 | | | | 6 | | 5 | | | 5 | | | | | 4 | | |  |  |
|  | MCL | 51 | | 51 | | | | 50 | | | 7 | | 7 | | | | 8 | | 2 | | 2 | | | | 2 | | 51 | | 51 | | | | 50 | | 7 | | 7 | | | | 8 | | 2 | | | 2 | | | | | 2 | | |  |  |
|  | LCL | 58 | | 58 | | | | 58 | | | 2 | | 2 | | | | 2 | | 0 | | 0 | | | | 0 | | 58 | | 58 | | | | 58 | | 2 | | 2 | | | | 2 | | 0 | | | 0 | | | | | 0 | | |  |  |
|  | ACL | 45 | | 45 | | | | 44 | | | 11 | | 11 | | | | 12 | | 4 | | 4 | | | | 4 | | 44 | | 45 | | | | 44 | | 12 | | 11 | | | | 12 | | 4 | | | 4 | | | | | 4 | | |  |  |
|  | PCL | 58 | | 58 | | | | 58 | | | 2 | | 2 | | | | 2 | | 0 | | 0 | | | | 0 | | 58 | | 58 | | | | 58 | | 2 | | 2 | | | | 2 | | 0 | | | 0 | | | | | 0 | | |  |  |
|  | | 0 | | | | | 1 | | | | | 2 | | | | | | 3a-c | | | | 4 | | | | | 0 | | | | | 1 | | | | 2 | | | | | | 3a-c | | | | | | 4 | | | | | | |  |
|  |  | Reader 1-3 | | | | | Reader 1-3 | | | | | Reader 1-3 | | | | | | Reader 1-3 | | | | Reader 1-3 | | | | | Reader 1-3 | | | | | Reader 1-3 | | | | Reader 1-3 | | | | | | Reader 1-3 | | | | | | Reader 1-3 | | | | | | |  |
| Cartilage defects | MFC | 37 | 38 | | 37 | | 1 | | 0 | | 1 | 6 | | 5 | | 6 | | 6 | 9 | 7 | | 10 | | 8 | | 9 | 37 | 37 | | 36 | | 0 | | 0 | 2 | 8 | | 8 | | 6 | | 6 | 6 | | 7 | | 9 | | | 9 | | 9 | |  |  |
|  | LFC | 46 | 47 | | 48 | | 3 | | 2 | | 0 | 5 | | 5 | | 5 | | 2 | 3 | 3 | | 4 | | 3 | | 4 | 47 | 47 | | 46 | | 2 | | 2 | 2 | 8 | | 8 | | 5 | | 1 | 1 | | 3 | | 2 | | | 2 | | 4 | |  |  |
|  | MTP | 41 | 42 | | 45 | | 4 | | 3 | | 1 | 7 | | 7 | | 5 | | 2 | 2 | 4 | | 6 | | 6 | | 5 | 42 | 42 | | 46 | | 4 | | 4 | 1 | 6 | | 6 | | 4 | | 2 | 2 | | 4 | | 6 | | | 6 | | 5 | |  |  |
|  | LTP | 51 | 50 | | 51 | | 1 | | 2 | | 1 | 3 | | 3 | | 3 | | 4 | 4 | 4 | | 1 | | 1 | | 1 | 49 | 49 | | 51 | | 2 | | 2 | 1 | 4 | | 4 | | 3 | | 4 | 4 | | 4 | | 1 | | | 1 | | 1 | |  |  |
|  | Trochlear groove | 45 | 46 | | 47 | | 4 | | 1 | | 0 | 3 | | 4 | | 4 | | 3 | 4 | 4 | | 5 | | 5 | | 5 | 43 | 43 | | 46 | | 4 | | 4 | 1 | 5 | | 5 | | 4 | | 2 | 3 | | 5 | | 6 | | | 5 | | 4 | |  |  |
|  | Retropatellar | 31 | 33 | | 31 | | 4 | | 2 | | 4 | 11 | | 9 | | 9 | | 8 | 10 | 10 | | 6 | | 6 | | 6 | 31 | 31 | | 32 | | 6 | | 6 | 4 | 9 | | 9 | | 8 | | 10 | 10 | | 10 | | 4 | | | 4 | | 6 | |  |  |
|  | | absent | | | | | | | | | | | | | present | | | | | | | | | | | | absent | | | | | | | | | | | | present | | | | | | | | | | | | | | | | |
|  |  | Reader 1-3 | | | | | | | | | | | | | Reader 1-3 | | | | | | | | | | | | Reader 1-3 | | | | | | | | | | | | Reader 1-3 | | | | | | | | | | | | | | | | |
| Bone marrow edema | Femoral | 43 | | | | 43 | | | | 43 | | | | | 17 | | | | 17 | | | | 17 | | | | 43 | | | | 43 | | | | 43 | | | | 17 | | | | | 17 | | | | | 17 | | | | | | |
|  | Tibial | 47 | | | | 47 | | | | 47 | | | | | 13 | | | | 13 | | | | 13 | | | | 47 | | | | 47 | | | | 47 | | | | 13 | | | | | 13 | | | | | 13 | | | | | | |
|  | Patellar | 48 | | | | 48 | | | | 48 | | | | | 12 | | | | 12 | | | | 12 | | | | 48 | | | | 48 | | | | 48 | | | | 12 | | | | | 12 | | | | | 12 | | | | | | |
| Fracture |  | 55 | | | | 55 | | | | 55 | | | | | 5 | | | | 5 | | | | 5 | | | | 55 | | | | 55 | | | | 55 | | | | 5 | | | | | 5 | | | | | 5 | | | | | | |
| Joint effusion |  | 19 | | | | 19 | | | | 18 | | | | | 41 | | | | 41 | | | | 42 | | | | 22 | | | | 22 | | | | 21 | | | | 38 | | | | | 38 | | | | | 39 | | | | | | |
| MCL indicates medial collateral ligament; LCL, lateral collateral ligament; ACL, anterior cruciate ligament; PCL, posterior cruciate ligament; MFC, medial femoral condyle; LFC, lateral femoral condyle; MTP, medial tibial plateau; LTP, lateral tibial plateau. | | | | | | | | | | | | | | | | | | | | | | | | | | | | | | | | | | | | | | | | | | | | | | | | | | | | |  |  |  |
